# A Novel Domain Adaptation-Based Intelligent Fault Diagnosis Model to Handle Sample Class Imbalanced Problem

**DOI:** 10.3390/s21103382

**Published:** 2021-05-12

**Authors:** Zhongwei Zhang, Mingyu Shao, Liping Wang, Sujuan Shao, Chicheng Ma

**Affiliations:** 1 School of Transportation and Vehicle Engineering, Shandong University of Technology, Zibo 255000, China; zhangzz@sdut.edu.cn (Z.Z.); ssjsdut@sdut.edu.cn (S.S.); machch@sdut.edu.cn (C.M.); 2 School of Mathematics and Information Science, Nanjing Normal University of Special Education, Nanjing 210038, China; wlp8631@nuaa.edu.cn

**Keywords:** fault diagnosis, samples class imbalance, manifold regularization, maximum variance discrepancy, domain adaptation

## Abstract

As the key component to transmit power and torque, the fault diagnosis of rotating machinery is crucial to guarantee the reliable operation of mechanical equipment. Regrettably, sample class imbalance is a common phenomenon in industrial applications, which causes large cross-domain distribution discrepancies for domain adaptation (DA) and results in performance degradation for most of the existing mechanical fault diagnosis approaches. To address this issue, a novel DA approach that simultaneously reduces the cross-domain distribution difference and the geometric difference is proposed, which is defined as MRMI. This work contains three parts to improve the sample class imbalance issue: (1) A novel distance metric method (MVD) is proposed and applied to improve the performance of marginal distribution adaptation. (2) Manifold regularization is combined with instance reweighting to simultaneously explore the intrinsic manifold structure and remove irrelevant source-domain samples adaptively. (3) The *ℓ*2-norm regularization is applied as the data preprocessing tool to improve the model generalization performance. The gear and rolling bearing datasets with class imbalanced samples are applied to validate the reliability of MRMI. According to the fault diagnosis results, MRMI can significantly outperform competitive approaches under the condition of sample class imbalance.

## 1. Introduction

Bearings and gears are vital components and are widely utilized in machinery equipment [1]. In addition, bearing and gear faults are the most common failure mode which may lead to unexpected fatal failures and elevated maintenance costs. Thus, there is a strong demand for intelligent fault diagnosis techniques of bearings and gears to ensure the security and reliability of mechanical equipment [2,3,4].

For deep learning methods, for instance, deep belief networks [5], sparse filtering [6], and autoencoders (AEs) [7,8], the main assumption is that the datasets applied to train and test the model have the same feature distribution. Unfortunately, the raw vibration signals are usually obtained under variable working cases in practical applications, which show deviation from the assumption [9,10]. As a result, poor performances may be obtained for most machine learning methods. The above issue is often denoted as cross-domain learning.

Within the last decade, DA techniques have been focused on solving the above problem. The source and target domain data show similar but different feature distributions for DA [11]. Many existing DA approaches usually aim to reduce the difference of cross-domain feature distributions, e.g., the distribution adaptation, the instance reweighting, or joint matching (join the distribution adaptation and instance reweighting). The distribution adaptation approaches [12,13,14] mainly include marginal adaptation (MDA) [15,16,17,18,19], conditional adaptation (CDA) [20], or both [12,21] and are applied for most distribution adaptation approaches. Lu et al. [15] adapted the marginal distribution by MMD to minimize the distribution discrepancy across domains and introduced MMD into a deep neural network (DNN). Han et al. [21] introduced the joint distribution adaptation (JDA) into a deep transfer network (DTN) to avoid negative adaptation and presented smooth convergence for fault diagnosis in industry applications. The discussion in [12] showed that joint distribution adaptation may obtain better performance in fault diagnosis by reweighting the source instance on the basis of its correlation with the target instance to reduce the cross-domain feature distribution discrepancy [22,23]. Chen et al. [23] developed an unsupervised domain adaptation approach to reduce the domain shifts between the data gathered from the experimental platform and the operating platform of the rotating machine by aligning the features extracted from the two data domains. In addition, some published DA methods tried to join feature reweighting and subspace learning [24,25]. Long et al. [24] reduced the cross-domain distribution discrepancy and achieved good classification results by combining these two learning strategies. However, the above approach matches the sample moments among distinct data distributions and down-weighs the irrelevant source domain features, which may perform badly while the data distribution discrepancy across the two domains is rather large, e.g., the sample class imbalance case.

Sample class imbalance denotes a situation where the number of instances in one class is much different from the number of instances in other classes. The class imbalance will lead to a substantially large cross-domain distribution difference and usually exists in many domain adaptation scenarios. Unfortunately, the class imbalance is usually ignored for most DA approaches [12,14]. They usually assume the sample classes are balanced or tackle the sample bias for one domain, which decreases the validity of DA. When the proportion of different classes is substantially imbalanced, distribution adaptation only or independent manifold learning is not enough to obtain good fault classification results. Thus, it is an important challenge to tackle the class imbalanced case in domain adaptation.

To this end, it is very necessary to study the deep information in the marginal distributions [26]. As a distance metric, MVD is very suitable for the class imbalance situation. In addition, manifold regularization can search the intrinsic manifold structure and further exploit the marginal distributions across domains. This motivates us to combine manifold regularization with the MVD, which can further extract effective information by optimizing the manifold consistency underlying marginal distributions and the manifold geometric structure. Moreover, the instance reweighting approach can further reduce the cross-domain difference by down-weighting the irrelevant source domain instances compared with target domain instances.

In recent years, manifold learning has drawn much attention in the field of fault diagnosis [27,28,29]. Wang et al. [27] applied manifold alignment for cross-domain fault diagnosis and decreased the distributional shift and structural shift at the same time via transforming the fault features into two low-dimension subspaces. Wang et al. [28] applied manifold learning to decrease the dimension of a wave pocket envelope matrix to learn the embedded inherent defect characteristics, and reveal the inherent envelope structure of impact impulses without the optimal band selection. Compared with the previous approaches, our work aims to model the manifold regularization, MVD and the instance reweighting techniques in a unified way to solve the class imbalance problem in fault diagnosis.

In this paper, considering the practical defect diagnosis application, a novel DA approach is proposed to handle the class imbalance problems. Firstly, the raw vibration signal under different rotating speed and load conditions are preprocessed by the fast Fourier transformation (FFT) to obtain the frequency spectrum. Then, ℓ2-norm regularization is applied for processing the frequency spectrum, which can improve the model generalization performance. Next, manifold regularization is combined with MVD and instance reweighting to simultaneously reduces the cross-domain distribution difference, geometric difference, and the proportion of unrelated source-domain samples, which can obtain the domain-invariant fault features with sufficient transferability. Finally, softmax regression is applied for predicting the fault types. Moreover, the fault features are normalized by the *z*-score normalization before fault classification to ensure the robustness of MRMI. The experimental results show that MRMI outperforms baseline DA approaches significantly.

The rest of this paper is organized as follows: In Section 2, DA, MMD, and the softmax regression algorithm are briefly presented. The framework of MRMI is described in Section 3. In Section 4, the validity and robustness of MRMI are validated according to the fault diagnosis experiments. Finally, the conclusions are given in Section 5.

## 2. Theoretical Background

### 2.1. Domain Adaptation

**Figure 1:**
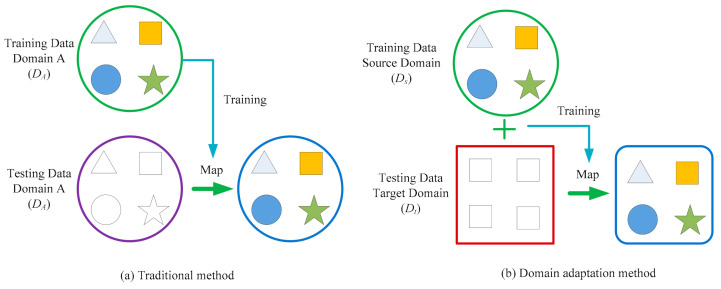
Intelligent learning system [13].

As we can see from Figure 1, the categories of data are represented by different shapes, and the labeled training data and test data have an identical data distribution for the traditional intelligent method. By contrast, for domain adaptation, the labeled source domain data $D_{s}=\left\{\left(x_{s_{1}},y_{s_{1}}\right),\cdots ,\left(x_{s_{ns}},y_{s_{ns}}\right)\right\}$ and unlabeled target domain data $D_{t}=\left\{x_{t_{1}},\cdots ,x_{t_{nt}}\right\}$ show the different but similar data distributions. The further description of domain adaptation is discussed as follows.

1. X refers to data space and *P* denotes a marginal data distribution. Thus, $\left\{X,P\left(X\right)\right\}$ denotes that dataset *X* is drawn from X and shows the data distribution $P\left(X\right)$. For DA, datasets have distinct data spaces and marginal data distributions, i.e., $X_{s}\neq X_{t}$ and *P_s_*(*X_s_*) ≠ *P_t_*(*X_t_*);
2. For the task $T=\left\{Y,f\left(X\right)\right\}$, the prediction function $f\left(X\right)=P\left(Y|X\right)$ denotes the conditional distribution and *Y* ∈ Y. $Y_{s}=Y_{t}$, $P\left(Y_{s}|X_{s}\right)=P\left(Y_{t}|X_{t}\right)$, where Y is the label spaces, since categories for distinct working conditions are the same.
3. In our research, a transfer function *F* is used to realize the domain adaptation learning, which satisfies $X_{s}\neq X_{t}$, $Y_{s}=Y_{t}$, $P(F\left(X_{s}\right)=P(F\left(X_{t}\right)$, and $P\left(Y_{s}|F\left(X_{s}\right)\right)=P\left(Y_{t}|F\left(X_{t}\right)\right)$.

### 2.2. Maximum Mean Discrepancy

The fundamental challenge for the generalization performance of DA approaches is to decrease the cross-domain distribution discrepancy. Thus, it is vital to minimize the discrepancy between cross-domain probability distributions by formalizing the distinct distribution and proposing effective approaches. Many parametric criteria have been applied to calculate the difference between cross-domain distributions, for instance, KL divergence [30] and Bregman divergence [31]. Nevertheless, as a more difficult density estimation process, the intermediate density estimate aggravates the model’s complexity. To solve this non-trivial problem, [32] ignored the intermediate density estimate, proposed a non-parametric divergence-MMD to compute the distance across domains by matching the data to the reproducing kernel Hilbert space (RKHS). Datasets *X* = {*x*_1_, ⋯, $x_{n_{1}}$} and *Y* = {*y*_1_, ⋯, $y_{n_{2}}$} obey the data distributions *P* and *Q*, respectively. The cross-domain distance is calculated as follows.

(1) $$Dist(X,Y)={\left\|\frac{1}{n_{1}}\sum_{i=1}^{n_{1}}f(x_{i})-\frac{1}{n_{2}}\sum_{i=1}^{n_{2}}f(y_{i})\right\|}_{H},$$

where H represents a universal RKHS [33], *φ*: *X*, *Y*→H.

### 2.3. Softmax Regression

The softmax regression (SR) model [34] has been widely used for the supervised learning stages of many domain adaptation approaches. Generally, the predicted labels of SR are multi-class classification instead of binary classification, so SR can be regarded as a generalized case for the logistic regression. In addition, SR is easy to carry out and it has high computing efficiency. To this end, the softmax regression classifier is selected for our research. It should be pointed out that the SR classifier is most suitable under the condition that the corresponding classes are mutually exclusive. Thus, we assume that each fault occurs separately.

The employed dataset is defined to train the softmax regression model, including *m* samples, that is, $\left\{\left(x^{1},y^{1}\right),\cdots \left(x^{m},y^{m}\right)\right\}$, where ***x***^(*i*)^ represents the input feature, and the labels consist of $y^{\left(i\right)}\in \left\{1,\;2,\cdots ,k\right\}$, where *k* represents the number of health conditions. Furthermore, *p*(***y***^(*i*)^ = *j*|***x***^(*i*)^) represents the probability value for which ***x***^(*i*)^ pertains to the category *j*. The probability value of each category is calculated for ***x***^(*i*)^, and then the output value is identified by selecting the category whose probability value is the maximum. Thus, the output value $h_{\theta }\left(x^{\left(i\right)}\right)$ can be written as:

(2) $$h_{\theta }\left(x^{\left(i\right)}\right)=\left[\begin{array}{c} p\left(y^{\left(i\right)}=1|x^{\left(i\right)};\theta \right)\\ p\left(y^{\left(i\right)}=2|x^{\left(i\right)};\theta \right)\\ \vdots \\ p\left(y^{\left(i\right)}=k|x^{\left(i\right)};\theta \right) \end{array}\right]=\frac{1}{\sum_{j=1}^{k}\exp\left(\theta_{j}^{T}x^{\left(i\right)}\right)}\left[\begin{array}{c} \exp\left(\theta_{1}^{T}x^{\left(i\right)}\right)\\ \exp\left(\theta_{2}^{T}x^{\left(i\right)}\right)\\ \vdots \\ \exp\left(\theta_{k}^{T}x^{\left(i\right)}\right) \end{array}\right],$$

where $\theta_{1},\theta_{2},\cdots \theta_{k}$ denote the parameters for the model.

The cost function $J\left(\theta \right)$ is displayed as follows:

(3) $$J\left(\theta \right)=-\frac{1}{m}\sum_{i=1}^{m}\sum_{j=1}^{k}1\left\{y^{\left(i\right)}=j\right\}\log\frac{\exp\left(\theta_{j}^{T}x^{\left(i\right)}\right)}{\sum_{l=1}^{k}\exp\left(\theta_{l}^{T}x^{\left(i\right)}\right)}+\frac{\lambda }{2}\sum_{i=1}^{k}\sum_{j=0}^{n}\theta_{ij}^{2},$$

where *m* represents the sample number, *n* refers to the *n*th column of weight matrix θ, *k* denotes category, λ is the weight decay term.

Generally, the cost function $J\left(\theta \right)$ is minimized by:

(4) $$\nabla_{\theta_{j}}J(\theta )=-\frac{1}{m}\sum_{i=1}^{m}\left[x^{\left(i\right)}(1\left\{y^{\left(i\right)}=j\right\}-p(y^{\left(i\right)}=j\left|x^{\left(i\right)};\theta \right.))\right],$$

$\nabla_{\theta_{j}}J\left(\theta \right)$ represents the partial derivative of $J\left(\theta \right)$ w.r.t. $\theta_{j}$, where j=1, 2,⋯k.

## 3. Proposed Framework

In this part, the data preprocessing for MRMI is firstly introduced in Section 3.1. Then, the model structure and the learning algorithm of MRMI are described in Section 3.2. In addition, Table 1 shows the frequently used notations.

### 3.1. Data Preprocessing

#### 3.1.1. Fast Fourier Transform (FFT)

First of all, FFT is adopted for transforming the original vibration signal into the frequency spectrum. The frequency spectrum can show the discrete frequencies of the constitutive components for the rotating machines [35] and can be good for extracting sensitive defect features that are easily discriminated.

#### 3.1.2. *ℓ*2-norm Regularization

Then, the *ℓ*2-norm regularization is adopted for the frequency spectrum to avoid the overfitting problem. The *ℓ*2-norm regularization can weaken the strong features as much as possible, and highlight the features with smaller values but more characteristics. Thus, it makes the corresponding algorithm more inclined to use all input features, rather than rely heavily on some parts of the input features, which may be very useful to calculate the similarity between two samples by the kernel methods. In general, the form of *ℓ*2-norm can be denoted as $\sqrt{{\left|t_{1}\right|}^{2}+\cdots +{\left|t_{n}\right|}^{2}}$, where $t=\left[t_{1},t_{2},\cdots ,t_{n}\right]$.

$f_{l}^{i}$ composes the data matrix, where *l* represents the row number and *i* is the column number. First of all, each row is regularized by the *ℓ*2-norm across all the samples.

(5) $$\bar{f_{l}}=f_{l}/\|f_{l}{\|}_{2},$$

Next, each column is regularized by its *ℓ*2-norm. As a result, the features lie on the unit *ℓ*2-ball.

(6) $$\hat{f}{}^{i}=\hat{f}{}^{i}/\|\hat{f}{}^{i}{\|}_{2},$$

Since the regularized features have been divided by their *ℓ*2-norm across all the samples, it means that the contributions of these features are almost the same.

#### 3.1.3. Data Dimensionality Reduction

As the most commonly used unsupervised linear dimensionality reduction approach, the principal component analysis (PCA) algorithm can map the high-dimensional vectors to the low-dimensional subspaces, and retain as much information as possible about the raw data. Thus, PCA is adopted for the dimensionality reduction of the regularized samples. As a result, the variance of the embedded data is maximized by the transformation matrix $U\in R^{m\times k}$.

(7) $$\underset{U^{T}U=I}{\max}tr\left(U^{T}XHX^{T}U\right),$$

where *tr* (·) denotes the matrix trace, $X=\left[x_{1},\cdots ,x_{n}\right]\in R^{m\times n}$ is the input matrix, $H=I-\frac{1}{n}1$ represents the centering matrix.

The kernel mapping form ψ: *x* ⟼ψ(*x*) and kernel matrix $K=\psi {\left(X\right)}^{T}\psi \left(X\right)\in R^{n\times n}$ are adopted for converting the data to RKHS. Then, the kernel-PCA is obtained by the representer theorem $V=\phi \left(X\right)A$.

(8) $$\underset{A^{T}A=I}{\max}tr\left(A^{T}KHK^{T}A\right),$$

where $A\in R^{n\times k}$ refers to the transformation matrix. As a result, the subspace embedding is transferred to $Z=A^{T}K$.

### 3.2. Model Structure and Learning Algorithm of MRMI

In this section, the model framework of MRMI is firstly presented, and then the corresponding learning algorithm is introduced.

#### 3.2.1. MRMI Model

The proposed MRMI is realized by minimizing the listed complementary objective functions:

(1) The MVD term for minimizing the discrepancy between the marginal probability distributions *P_s_* and *P_t_*;
(2) The *ℓ*2,1-norm structured sparsity regularization term for reweighting the source domain instances by structured sparsity;
(3) The manifold regularization for maximizing the manifold consistency between *P_s_* and *P_t_*.

The prediction function $f=w^{T}\phi \left(x\right)$ is applied for classification, where *w* denotes a parameter of the classifier. The final objective function of MRMI is summarized as follows.

(9) $$f=\;\arg\;\underset{f\in H_{K}}{\min}D_{f,K}\left(P_{s},P_{t}\right)+\lambda \;\|T{\|}_{2,1}+\gamma M_{f,K}\left(P_{s},P_{t}\right),$$

where $H_{K}$ denotes a set of *f* in the kernel space, *K* represents the kernel function which is calculated by ϕ, so $\langle \phi \left(x_{i}\right),\phi \left(x_{j}\right)\rangle =K\left(x_{i},x_{j}\right)$. In addition, the raw feature vector is projected into a Hilbert space H by the mapping function ϕ:X↦H [26]. *T* denotes the feature transformation to adapt different domains, $\|T{\|}_{2,1}$ represents the *ℓ*2,1-norm of *T*. $D_{f,K}\left(P_{s},P_{t}\right)$ denotes the discrepancy for *P_s_* and *P_t_*, and $M_{f,K}\left(P_{s},P_{t}\right)$ represents the manifold regularization which can extract more information from *P_s_* and *P_t_*. *λ* represents the regularization parameter which is employed for trading off instance reweighting and feature matching. *γ* refers to positive regularization parameters. Each term in Equation (9) is interpreted in the following discussion.

(1) *MVD Term*

While the distribution discrepancy across domains is rather large, the MMD algorithm performs badly for marginal distribution adaptation as MMD mainly regards the first-order statistics. By contrast, MVD simultaneously regards the first-order and second-order statistics, which shows better performance of marginal distribution adaptation and can bridge the cross-domain discrepancy more effectively than MMD. In addition, the deviation of cross-domain data distribution is reduced while the variance difference is decreased. For MRMI, we introduce MVD for the feature matching to further decrease the distribution difference.

In general, we can obtain the sample variance S^2^ by:

(10) $$S^{2}=\frac{\sum_{i=1}^{n}{\left(x_{i}-\bar{x}\right)}^{2}}{n},$$

where *n* represents the size of the sample, S denotes the standard deviation for the sample, and $\bar{x}$ is the average value.

In addition, the sample variance can be transferred into the other form DU.

(11) $$\mathrm{DU}=E\left(U^{2}\right)-{\left[E\left(U\right)\right]}^{2},$$

where U denotes a vector of sample, EU represents the expectation.

Let **Z***_i_* represents the *i*th sample of the subspace embedding, we can obtain:

(12) $$E\left(Z_{i}\right)=\sum_{i=1}^{n}f\left(Z_{i}\right)Z_{i},$$

where *f* (**Z***_i_*) denotes the probability value of the *i*-th sample.

The probability value that every sample occurred is assumed to be equal. As a result, Equation (12) can be calculated by:

(13) $$E\left(Z_{i}\right)=\frac{\sum_{i=1}^{n}Z_{i}}{n},$$

Kernel-PCA is applied to obtain the *k* dimension embedding for MVD. Then, the corresponding empirical mathematical expectations are computed by joining Equations (8) and (11).

(14) $$\begin{array}{c} \|\frac{1}{n_{s}}\sum_{i=1}^{n_{s}}{\left(A^{T}k_{i}\right)}^{2}-\frac{1}{n_{t}}\sum_{j=n_{s}+1}^{n_{s}+n_{t}}{\left(A^{T}k_{j}\right)}^{2}{\|}_{H}^{2}+\|\frac{1}{n_{s}}\sum_{i=1}^{n_{s}}A^{T}k_{i}+\\ \frac{1}{n_{t}}\sum_{j=n_{s}+1}^{n_{s}+n_{t}}A^{T}k_{j}{\|}_{H}^{2}\times \|\frac{1}{n_{s}}\sum_{i=1}^{n_{s}}A^{T}k_{i}-\frac{1}{n_{t}}\sum_{j=n_{s}+1}^{n_{s}+n_{t}}A^{T}k_{j}{\|}_{H}^{2}=\mathrm{tr}\left(A^{T}K_{1}MK_{1}{}^{T}A\right)-\\ \mathrm{tr}\left(A^{T}KMK^{T}A\right)*\mathrm{tr}\left(A^{T}KM_{1}K^{T}A\right), \end{array}$$

where $K_{1}=\psi {\left(X^{2}\right)}^{T}\psi \left(X^{2}\right)\in R^{n\times n}$, **M** and **M**_1_ are both the MVD matrix, which can be computed as follows

(15) $$M_{\mathrm{ij}}=\left\{\begin{array}{c} \frac{1}{n_{s}n_{s}},x_{i},x_{j}\in D_{s}\\ \frac{1}{n_{t}n_{t}},x_{i},x_{j}\in D_{t}\\ -\frac{1}{n_{s}n_{t}},\mathrm{otherwise} \end{array}\right.,M_{1\mathrm{ij}}=\left\{\begin{array}{c} \frac{1}{n_{s}n_{s}},x_{i},x_{j}\in D_{s}\\ \frac{1}{n_{t}n_{t}},x_{i},x_{j}\in D_{t}\\ \frac{1}{n_{s}n_{t}},\mathrm{otherwise} \end{array}\right.,$$

where $n_{s}$ and $n_{t}$ represent the samples of the source and target domain, respectively.

(2) *The ℓ2,1-norm Structured Sparsity Regularization Term*

Nevertheless, only applying the MVD term for minimizing $D_{f,K}\left(P_{s},P_{t}\right)$ is not enough to obtain representative features, because there are some irrelevant and redundant source instances. To this end, it is very necessary to down-weight the irrelevant source instances to further decrease domain discrepancy. In this section, we employ instance reweighting by the *ℓ*2,1-norm structured sparsity regularization to down-weight the irrelevant source instances in the instance space. The *ℓ*2,1-norm regularization is applied to induce *row-sparsity* in matrix ***A***. Owing to *row-sparsity*, each row of the transformation matrix ***A*** can be regarded as an instance which intrinsically facilitates the instance reweighting. Instance reweighting regularization can be constructed in the following way [24].

(16) $$\|A_{s}{\|}_{2,1}+\|A_{t}{\|}_{F}^{2},$$

where $A_{s}≔A_{1:n_{s},:}$ represents the source domain transformation matrix, and $A_{t}≔A_{n_{s}+1:n_{s}+n_{t},:}$ denote the target domain one. It should be noted that *ℓ*2,1-norm regularization is only employed to reweight the source domain instances with their correlation to the target ones. When Equation (16) is minimized, Equation (9) will be maximized, which means that the irrelevant and redundant source instances are down-weighted adaptively in a novel subspace embedding $Z=A^{T}K$. As a result, the robustness of MRMI is improved for the domain discrepancy resulting from irrelevant source instances.

(3) *Manifold Regularization Term*

The MVD term and the *ℓ*2,1-norm structured sparsity regularization term can reduce the domain discrepancy in H and the instance space, respectively. However, they only match the cross-domain sample moments and down-weight the irrelevant source domain features, which may perform badly when feature distribution discrepancy across domains is rather large, e.g., the class imbalance problem. Thus, manifold regularization is induced for researching the intrinsic manifold structure and further exploiting the information from *P_s_* and *P_t_* to learn better functions. Generally, the unlabeled target domain data may reveal the potential and hidden information, such as sample variances. According to the *manifold assumption* [36], the conditional distributions $Q_{s}\left(y_{s}|x_{s}\right)$ and $Q_{t}\left(y_{t}|x_{t}\right)$ are similar, when data points $x_{s},x_{t}\in X$ are close to each other in the geometry structure. After smoothing the geodesic, manifold regularization is calculated as

(17) $$M_{f,K}\left(P_{s},P_{t}\right)=\sum_{i,j=1}^{n_{s}+n_{t}}{\left(f\left(x_{i}\right)-f\left(x_{j}\right)\right)}^{2}W_{ij}=\sum_{i,j=1}^{n_{s}+n_{t}}f\left(x_{i}\right)L_{ij}f\left(x_{j}\right),$$

where ***W*** represents the graph affinity matrix, and ***L*** denotes the normalized graph Laplacian matrix. In addition, ***W*** is formulated as [37]

(18) $$W_{ij}=\left\{\begin{array}{cc} \cos\left(x_{i},x_{j}\right), & if\;x_{i}\in N_{p}\left(x_{j}\right)\vee \;x_{j}\in N_{p}\left(x_{i}\right)\\ 0, & \mathrm{otherwise} \end{array}\right.,$$

where $N_{p}\left(x_{i}\right)$ refers to the *p*-nearest neighbors. ***L*** can be calculated as $L=I-D^{-1/2}WD^{-1/2}$ [26].

Maximizing the consistency of the intrinsic manifold structure can be used to further explore the marginal data distributions via regularizing (14) with (17), and the discriminative hyperplanes across domains can be substantially matched. According to the representer theorem, the manifold regularization can be rewritten as

(19) $$M_{f,K}\left(P_{s},P_{t}\right)=tr\left(A^{T}KLK^{T}A\right),$$

Above all, by combining Equations (14), (16) and (19), the final objective function is obtained as follows:

(20) $$\underset{A^{T}KHK^{T}A=I}{\min}tr\left(A^{T}K_{1}MK_{1}{}^{T}A\right)-tr\left(A^{T}KMK^{T}A\right)*tr\left(A^{T}KM_{1}K^{T}A\right)+\lambda \left(\|A_{s}{\|}_{2,1}+\|A_{t}{\|}_{F}^{2}\right)+\gamma \left(tr\left(A^{T}KLK^{T}A\right)\right),$$

by regarding ***A*** as the adaptation matrix throughout the rest of this article to emphasize its functionality. It provides great convenience for the implementation and deployment of MRMI because a principled dimensionality reduction procedure is applied.

(4) *Construct the Softmax Regression Classifier*

In the process of classification, *z*-score normalization can eliminate the influence of dimension on classification results to develop the classification accuracy. Moreover, the learning rate and the efficiency of dealing with the optimal solution in the process of back propagation can be optimized via *z*-score normalization. Hence, it is adopted for processing the input data for the classifier. In other words, the training data ***T_r_*** and the testing data ***T_t_*** are computed by ***T_r_*** = F(***Z_S_***) and ***T_t_*** = F(***Z_T_***), where ***Z_S_*** = $A_{s}^{T\;}K_{s}$ and ***Z_T_*** = $A_{t}^{T\;}K_{t}$. *Z*-score normalization is formulated as follows:

(21) $$F\left(X\right)=\frac{X-\bar{X}}{\sigma },$$

where ***X*** denotes invariant feature subspace ***Z_S_*** or ***Z_T_*** in the finite domain, $\bar{X}$ refers to the average value of ***X***, σ is the standard deviation. After carrying out *z*-score normalization, the rescaled subspace $F\left(X\right)$ with a standard normal distribution is acquired.

Then, the probability value $p(y^{\left(i\right)}=j|T_{t}^{\left(i\right)})$ corresponding to each category *j* is calculated by Equation (2), then the fault category is predicted by selecting the *j* with maximum value. Finally, the classification performance of MRMI is obtained by comparing the consistency between the predicted fault type and the real one.

#### 3.2.2. Learning Algorithm

By the constrained optimization theory, $\Phi =\mathrm{diag}\left(\phi_{1},\cdots ,\phi_{k}\right)\in R^{k\times k}$ is adopted as the Lagrange multiplier for Equation (20). Thus, the Lagrange function is derived as:

(22) $$F=tr\left(A^{T}K_{1}MK_{1}{}^{T}A\right)-tr\left(A^{T}KMK^{T}A\right)*tr\left(A^{T}KM_{1}K^{T}A\right)+\lambda \left(\|A_{s}{\|}_{2,1}+\|A_{t}{\|}_{F}^{2}\right)+\gamma tr\left(A^{T}KLK^{T}A\right)+tr(\left(I-A^{T}KHK^{T}A\right)\Phi ,$$

Let $\frac{\partial F}{\partial A}=0$, generalized eigen-decomposition is approximately calculated as:

(23) $$\left(K_{1}MK_{1}{}^{T}-\left(KMK^{T}\right)*\left(KM_{1}K^{T}\right)+\lambda G+\gamma KLK^{T}\right)A=KHK^{T}A\Phi ,$$

As $\|A_{s}{\|}_{2,1}$ refers to a non-smooth function, the subgradient is computed as $\frac{\partial \left(\|A_{s}{\|}_{2,1}+\|A_{t}{\|}_{F}^{2}\right)}{\partial A}=2GA$, where ***G*** represents a diagonal subgradient matrix which consists of the *i*-th element as below:

(24) $$G_{ii}=\left\{\begin{array}{c} \frac{1}{2\|\alpha^{i}\|},\;\;\;\;x_{i}\in D_{s},\alpha^{i}\neq 0\\ 0,\;\;\;\;\;\;\;\;\;\;\;\;x_{i}\in D_{s},\alpha^{i}\neq 0\\ 1,\;\;\;\;\;\;\;\;\;\;\;\;\;\;\;\;\;\;\;\;\;\;\;x_{i}\in D_{t} \end{array}\right.,$$

In the next step, matrix ***A*** is reduced to *k* smallest eigenvectors by (23). Nevertheless, the subgradient matrix ***G*** and adaptation matrix ***A*** are not known in advance. To overcome this deficiency, the parameters are optimized alternately by updating one parameter while fixing the other one.

**Figure 2:**
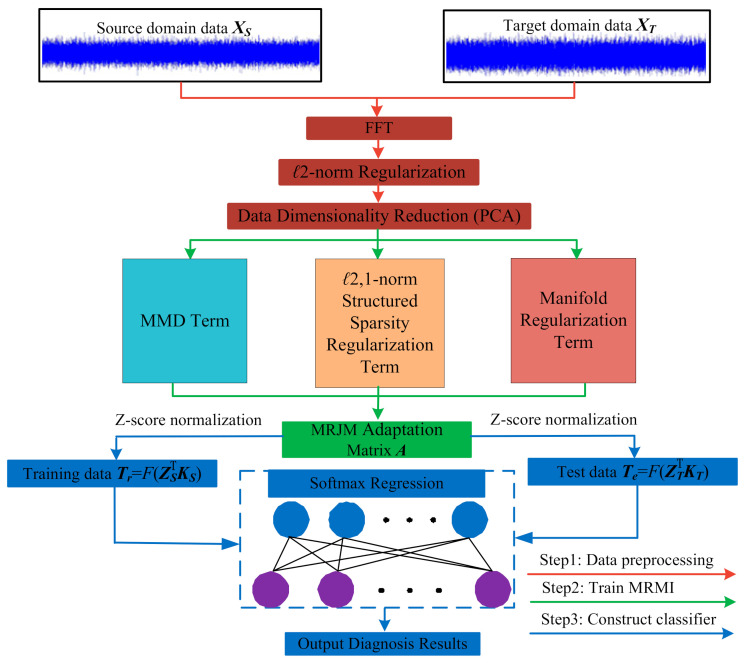
The framework of MRMI.

For better interpretation, the structure of MRMI is shown in Figure 2.

## 4. Experiment Results and Analysis

### 4.1. Case 1: Bearing Fault Diagnosis

#### 4.1.1. Experimental Setup and Data Description

A rolling bearing dataset offered by Case Western Reserve University was employed to validate the performance of MRMI in this part [38]. It was acquired by accelerometers installed in the driving position of the motor and includes the normal (Nor) and faulty data. Furthermore, the faulty data consist of a single-point fault at the inner bearing race (FI), the outer race (FO), and the roller (FR). Each defect type of the faulty dataset contains three fault levels, i.e., 0.18, 0.36, 0.54 mm. Therefore, there are 10 health types obtained for the rolling bearing dataset in this section. The vibration signals were acquired under four loads (0, 1, 2, 3 hp). In addition, the sampling frequency was fixed as 12 kHz. In addition, we select the four motor loads as the four scenarios for domain adaptation. To simulate the situation of class imbalance, Table 2 shows the sample distribution for all domain adaptation tasks.

In Table 2, the vibration data collected with load 0, 1, 2, 3 hp are chosen as the DA scenarios A, B, C, D, respectively. The numbers of experimental samples for source and target domains are distinct from each other for different DA scenarios. In DA task “B→D”, B represents the labeled source domain dataset which includes 205 experimental samples collected under load 1 hp, while D denotes the unlabeled target domain dataset which contains 1000 experimental samples collected with load 3 hp. Therefore, the data distributions of these two domains are imbalanced.

First of all, the data preprocessing process is conducted for the rolling bearing dataset. As a result, the spectra of original vibration signals are obtained by fast Fourier transformation (FFT). Then, the time-domain samples with 1200 sample lengths are converted to 600 length samples in the frequency domain.

#### 4.1.2. Experimental Results

(1) *Comparison Methods*

To validate the effectiveness of manifold regularization-based joint matching (MRMI), several successful domain adaptation approaches are selected as the baseline methods. The details of these baseline approaches are described as follows.

1. Deep neural network (DNN)-based DA approach (DAFD) [35], which combines MMD with DNN to extract the domain-invariant features;
2. Geodesic flow kernel (GFK) [15,18], which represents a typical DA approach;
3. Transfer joint matching (TJM) [24], which introduces feature matching into instance reweighting;
4. Adaptation regularization-based transfer learning (ARTL) [26], which combines JDA with manifold regularization;
5. Domain-adversarial neural networks (DANNs) [39], which develop a novel representation learning method for DA.

(2) *Setup of the Algorithm*

To provide a relatively fair environment for comparison, the hyperparameter space is empirically searched to select the best parameter settings. For reducing the randomness of the experiments, we carry out 15 trials of experiments to every DA task, then calculate the average classification accuracy to evaluate the performance for each approach. Moreover, the SR classifier is adopted for predicting the fault types of the target domain for all these domain adaptation methods.

For all the baseline approaches, the optimum dimension of the subspace is obtained by searching {10, 20, ⋯, 200} and the optimum value is selected by searching {0.001, 0.01, 0.1, 1, 10, 100, 1000}. In addition, the structure of the neural network is {600, 1000, 10] for DAFD [37], and the size of the hidden layer for DANN is set as 200.

The proposed method contains only three model parameters: subspace dimension *k*, regularization parameters λ and γ. Empirical analysis of parameter sensitivity will be discussed in a later section. According to the parameter selection of the baseline approaches, the parameters of MRMI are set as *k* = 50, λ = 1, γ = 10 and the linear kernel is employed for MRMI.

In this paper, the diagnosis accuracy for the unlabeled data of the target domain is employed as the performance evaluation index, which has been applied in numerous published studies [40,41,42].

(25) $$CA=\frac{\left|x:x\in D^{t}⋀\;\mathrm{prediction}\left(y\right)=\mathrm{true}\left(y\right)\right|}{\left|x:x\in D^{t}\right|}\times 100\%,$$

(3) *Results*

For the experiment in this section, 12 DA scenarios are selected: A→B, A→C, A→D, B→A, B→C, B→D, C→A, C→B, C→D, D→A, D→B, and D→C. The experimental results of MRMI and all baseline methods are illustrated in Table 3. The result shows that the average accuracy of DA task a→b is distinct from b→a, e.g., the classification result of A→D is 99.50% for MRMI but is 96.86% for scenario D→A.

As we can see from the results listed in Table 3, MRMI yields the best diagnosis accuracy and robustness and outperforms the other four listed compared approaches in most (11 out of 12) domain adaptation scenarios. This indicates that more transferable and robustness fault features could be extracted for MRMI. Furthermore, we can draw several observations as follows.

Firstly, the proposed method performs worse than GFK in the scenario D→A. For GFK, the final diagnosis result for all 12 domain adaptation tasks can reach 90.91% which is the highest accuracy compared with the other baseline approaches and is 8.55% less than MRMI. The smooth transmission of the object datasets can be guaranteed by mapping the global GFK into a low dimension representation, thus, good diagnosis performance can be obtained. Nevertheless, GFK performs worse in DA scenarios A→D and B→D, which indicates that only applying the geodesic flow distance to correct the distribution mismatch is not enough when the cross-domain discrepancy is rather large.

Secondly, DAFD combines MMD with DNN to extract the domain-invariant features. However, DAFD performs worse than MRMI, which highlights that MVD can bridge the cross-domain difference more effectively than MMD. The reason is that MVD simultaneously regards the first-order and second-order statistics to minimize the marginal distribution mismatch. In addition, from the results, we also observe that only adopting the marginal distribution adaptation is not enough to reduce the cross-domain conditional distribution difference. Therefore, the average classification accuracy for DAFD is under 80%, which performs worse than ARTL and TJM.

Thirdly, TJM combines instance reweighting with MMD in a principled dimensionality reduction process to reduce the cross-domain discrepancy. In addition, TJM aims to build a novel feature representation. It is invariant to distribution discrepancy and irrelevant source instances. Thus, TJM performs well when the cross-domain distribution difference is rather large. However, MMD mainly regards the first-order statistics, and while the distribution discrepancy across domains is rather large, the MMD algorithm performs badly for marginal distribution adaptation. As a result, the average classification accuracy is still 16.06% lower than the proposed approach, which indicates that information of *P_s_* and *P_t_* needs to be further explored to extract more representative transferable features for TJM.

Fourthly, MRMI significantly outperforms ARTL, which is a state-of-the-art DA approach based on JDA and manifold regularization. ARTL only matches the features without reweighting source instances. As a result, when cross-domain distribution discrepancy is larger, some source instances which are irrelevant to the target instances will always be contained in the feature-matching subspace. Thus, compared with ARTL, the performance boost of 12.32% can be achieved for MRMI.

Finally, the average accuracy for DANN can reach 89.80%, which performs worse than the proposed approach on the whole. In particular, for the DA tasks A→D and B→D, the accuracies of DANN can only reach 73.90% and 65.70%, respectively. This indicates that the performance of DANN decreases dramatically when the cross-domain discrepancy is substantially large.

#### 4.1.3. Effectiveness Analysis

(1) *Feature Distribution*

**Figure 3:**
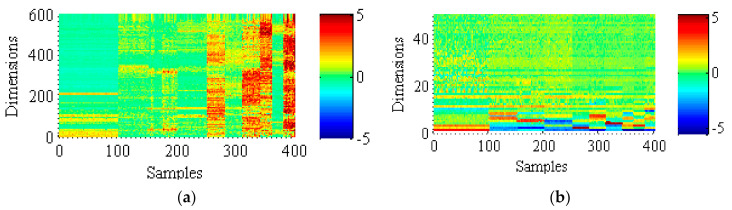
Feature distributions of the unlabeled target domain data based on the learned transferable features (DA task B→C): (**a**) GFK; (**b**) MRMI.

The distribution of features drawn by GFK and MRMI for domain adaptation scenario B→C is displayed in Figure 3. It can be seen from Figure 3 that the abscissa denotes a total of 400 samples and the amount of samples contained in different fault types is imbalanced. Furthermore, the ordinate represents the dimensions of each sample and different colors refer to the different amplitude sizes. According to the feature distributions extracted by GFK, many defect features are identified. However, some fault features still perform similarly. For MRMI, the discrepancies among distinct defect feature distributions are more obvious which makes the fault category easier to be distinguished. Thus, MRMI can extract more discriminative and representative features and obtain better classification performance.

(2) *Discussion for MRMI*

**Figure 4:**
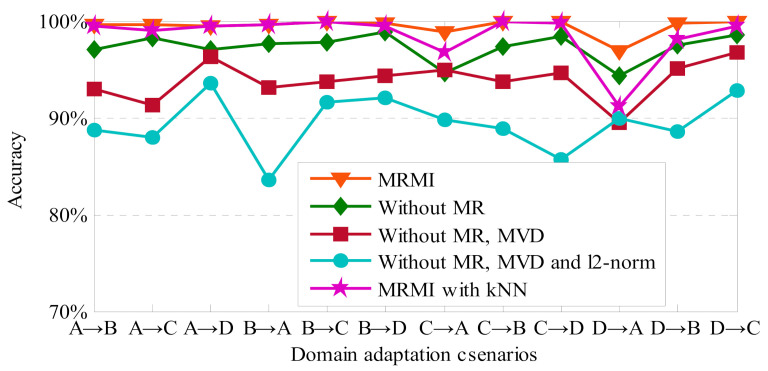
Classification results of single-factor experiments for MRMI.

MRMI greatly outperforms the baseline approaches mainly by introducing *ℓ*2-norm regularization, manifold regularization, MVD, and instance reweighting. Several single-factor-based experiments are executed to further study the contributions of these components for MRMI individually, and the experimental results are depicted in Figure 4. To further show the effectiveness of the components of the proposed model, the results of the ablation study for MRMI are summarized in Table 4. Based on the ablation study, it can be seen that the average diagnosis accuracy of MRMI without manifold regularization (MR) can reach 97.28%, which is 2.18% lower than MRMI. This indicates that inducing manifold regularization can obtain a 2.18% transfer improvement comparing with MRMI without MR. For the proposed method, when we do not apply manifold regularization and MVD, the average classification accuracy is 93.85%. This result means that the contribution of MVD to the diagnosis accuracy of MRMI is 3.43%. For MRMI, when we do not apply manifold regularization, MVD, and *ℓ*2-norm, the average classification accuracy is 89.41%. This result means that only inducing *ℓ*2-norm can bring a 4.44% accuracy improvement for the proposed method. When k-nearest neighbor (kNN) is applied as the classifier for MRMI, the final diagnosis result is 0.94% lower than the proposed approach which can reach 98.52%. Notably, the accuracy for task D→A of MRMI with kNN is only 91.2%, which indicates the bad robustness of the kNN classifier in this experiment. Thus, the softmax regression classifier-based MRMI can obtain better diagnostic performance than the kNN classifier-based one.

Moreover, according to the experiment results, it is necessary to join *ℓ*2-norm regularization, manifold regularization, MVD, and instance reweighting to guarantee the effectiveness and robustness of MRMI while the distribution difference is rather large.

(3) *Confusion Matrix*

**Figure 5:**
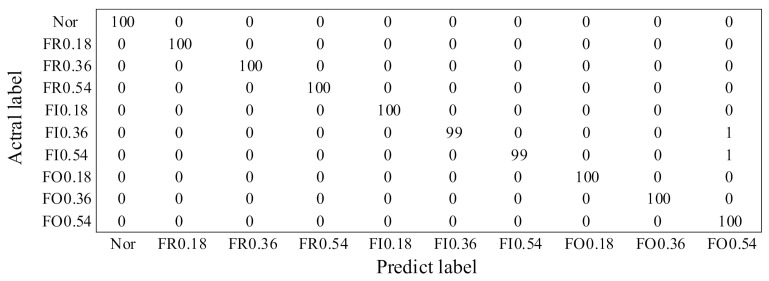
Confusion matrix of the fault diagnosis results for DA task B→D.

To further study the fault diagnosis effectiveness of MRMI, the confusion matrix of the classification results for DA scenario B→D is displayed in Figure 5. In Figure 5, the rows represent the actual defect types, and the columns stand for the predicted defect types. As we can see from Figure 5, the misclassification issue mainly happens for the defect types of FI 0.36 and FI 0.54. In detail, only one sample of FI 0.36 and one sample of FI 0.54 are misclassified to FO 0.54, thus the classification accuracy of 99.8% is finally obtained for domain adaptation task B→D.

(4) *Feature Visualization*

**Figure 6:**
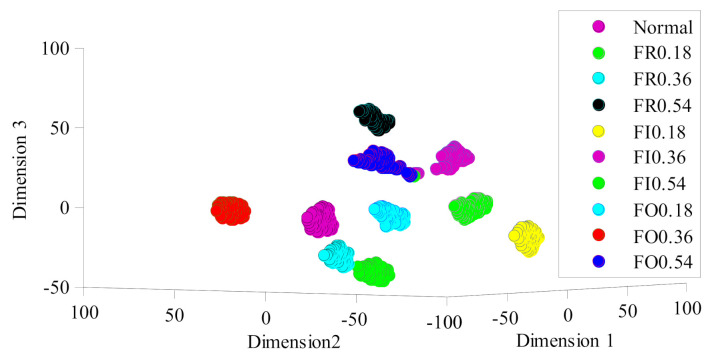
Visualization maps of the learned features of DA task B→C.

In this section, we execute the t-SNE [43] algorithm to transform the 100-dimension feature vector into a map with 3 dimensions to estimate the ability to learn representative features for MRMI. For instance, the visualization maps of MRMI for DA task B→C is built, and the results are depicted in Figure 6. We can see that most fault features with the same labels are concentrated in the corresponding cluster and different clusters are separated from each other [37]. Thus, MRMI is verified to show strong feature learning ability.

(5) *Parameter Sensitivity*

**Figure 7:**
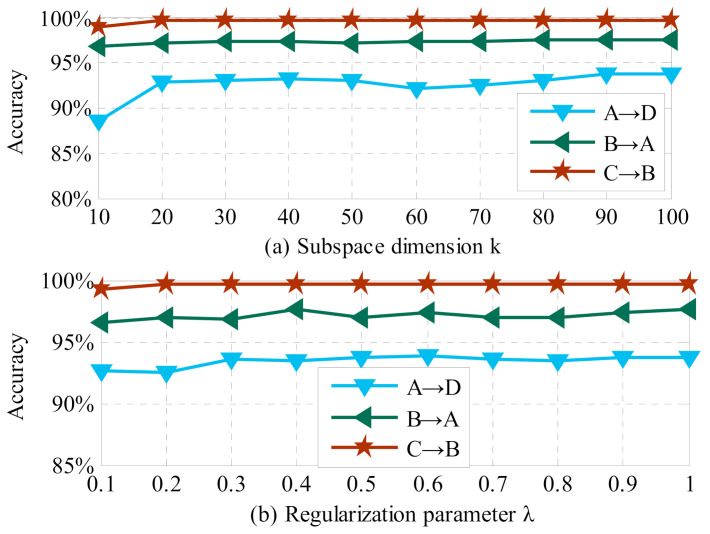

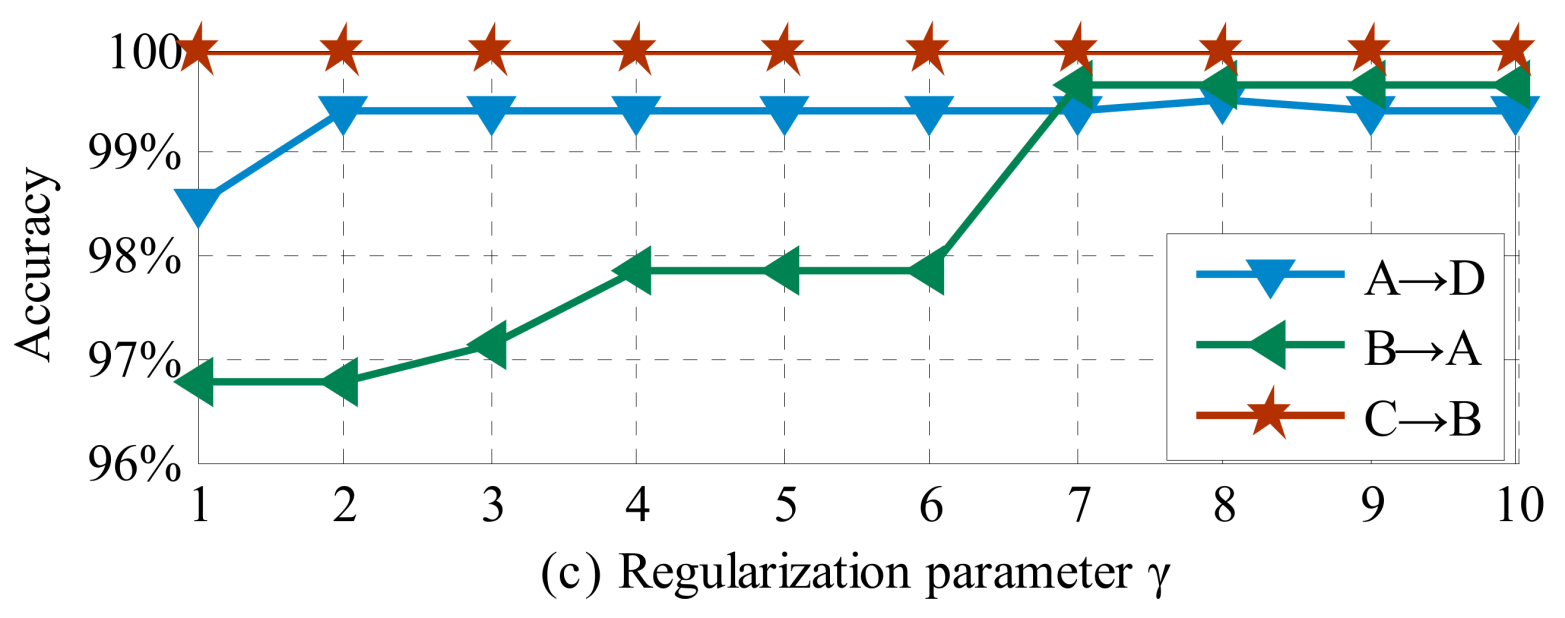
Parameter sensitivity for MRMI on rolling bearing datasets.

In this part, sensitivity analysis on representative DA tasks A→D, B→A, and C→B is employed for evaluating the effectiveness and selection of the parameters for MRMI due to space limitation. The classification results with respect to varied parameters *k*, λ, and γ are displayed in Figure 7. First of all, we implement MRMI with varied values of *k* ∈ [10, 100], and the other parameters are fixed as λ = 1 and γ = 10. According to the results shown in Figure 7a, stable classification performances can be obtained when subspace dimension k is larger than 50. Thus, we select *k* ∈ [50, 100] for MRMI. Then, the proposed approach with varying values of λ ∈ [1, 10] is executed when *k* = 50 and γ = 10. From Figure 7b, robust diagnosis accuracies can be gained with λ ∈ [3, 6]. Finally, varying values of regularization parameter γ∈ [1, 10] are implemented for MRMI with the other parameter settings of *k* = 50 and λ = 1. As we can see from Figure 7c, stable diagnosis performance is obtained when γ is larger than 7. Therefore, the optimum regularization parameter γ is set as γ∈ [7, 10].

### 4.2. Case 2: Gear Fault Diagnosis

#### 4.2.1. Experimental Setup and Data Description

To further verify the effectiveness of MRMI, a gear dataset with different loads provided by a specially designed gearbox platform is adopted in this part [44]. The raw signals of gears were collected by the sensors installed on the fixed plate of the driving end. Four types of gear fault are considered for the gear fault diagnosis experiment: (1) Single wheel pitting fault; (2) single pinion wear fault; (3) compound fault of pinion wear and wheel pitting; (4) compound fault of pinion wear and wheel teeth broken. We define the normal state and these four kinds of faults as Type 1 to Type 5, respectively. In addition, the raw vibration signal was acquired with three distinct loads which were denoted as dataset A, B, and C, respectively. 

The same as the rolling bearing experiment in case 1, a class imbalanced dataset is adopted for the gear fault diagnosis experiment. The distribution of each dataset is illustrated in Table 5. In addition, the original samples of each dataset are selected alternately to avoid overlap between samples. Then, FFT is employed for preprocessing the raw data. Finally, the time-domain sample containing 1200 datapoints is converted to the frequency-domain sample containing 600 data points.

#### 4.2.2. Experimental Results

**Figure 8:**
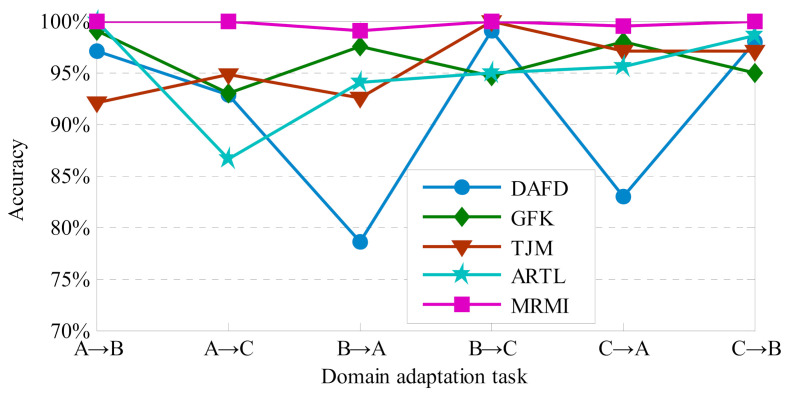
The diagnosis results for sample class imbalanced gear dataset.

In this experiment, the compared methods and their corresponding parameter selection method are the same as those of the experiment in case 1. Furthermore, six DA scenarios are adopted for empirical evaluation: B→A, B→C, C→A, C→B, A→B, and A→C. The fault classification results for MRMI and the compared methods are displayed in Figure 8.

It can be seen from Figure 8 that MRMI significantly outperforms the listed baseline approaches in all the domain adaptation scenarios.

Since only six fault types are included in each gear dataset, and the discrepancy between cross-domain distributions is small, higher classification levels can be gained for the DA approaches. Thus, the diagnosis results of all the approaches depicted in Figure 7 are all over 90%. The mean classification result of the six DA tasks can reach 99.75%, and a 3.57% diagnosis performance improvement is acquired for MRMI in comparison to GFK which can obtain the best diagnosis performance among all baseline methods. In general, DAFD performs worse than the other baseline approaches, especially in the DA scenarios B→A and C→A. TJM and ARTL can acquire good classification results, and their mean accuracies are only 4.2% and 4.8% lower than that of MRMI, respectively. Moreover, the robustness of MRMI also performs better than the other compared methods according to the diagnosis results. All in all, the classification results of the gear dataset prove the effectiveness and robustness of MRMI.

## 5. Conclusions

This study develops a new MRMI method for mechanical fault diagnosis in a class imbalance environment. MRMI joins manifold regularization, MVD, and instance reweighting to handle the class imbalance problem. In addition, *ℓ*2-norm regularization is employed for improving the generalization ability of MRMI. The proposed method is tested on two sample class imbalanced vibration datasets. The classification results show that MRMI can effectively extract more transferable features and significantly outperform the other four baseline domain adaptation approaches while the distribution discrepancy across domains is rather large. Thus, MRMI is a robust and effective DA model for cross-domain mechanical fault diagnosis problems. In the near future, MRMI could be extended to other related fields, such as online health monitoring.

## Figures and Tables

**Table 1:** Notations and descriptions.

Notation	Description	Notation	Description
*D_s_*, *D_t_*	Source/Target domain	***X***	Input data matrix
*n_s_*, *n_t_*	Source/Target samples	***A***	Alignment matrix
$X_{s}$, $X_{t}$	Source/Target data space	***L***	Laplacian matrix
*k*	Subspace bases	***M***	MMD matrix
λ, γ	Regularization parameter	***G***	Subgradient matrix
***Z***	Subspace embedding	***K***	Input kernel matrix

**Table 2:** Rolling bearing dataset with sample class imbalance distribution.

Fault Location	Nor	Roller			Inner Ring			Outer Ring			Total
Category Labels	1	2	3	4	5	6	7	8	9	10	
Fault Size (mm)	0	0.18	0.36	0.54	0.18	0.36	0.54	0.18	0.36	0.54	
A (load 0)	100	30	20	10	30	20	10	30	20	10	280
B (load 1)	100	10	15	10	10	15	10	10	15	10	205
C (load 2)	100	50	50	50	30	30	30	20	20	20	400
D (load 3)	100	100	100	100	100	100	100	100	100	100	1000

**Table 3:** The classification results (%) on class imbalanced rolling bearing dataset.

Source Domain	Method	Target Domain			
		A	B	C	D
A	DAFD	-	81.46 ± 1.21	83.25 ± 0.36	71.80 ± 0.60
	GFK	-	96.10 ± 0.15	88.25 ± 0.00	78.20 ± 0.60
	TJM	-	88.78 ± 0.49	76.00 ± 0.25	95.6 ± 1.10
	ARTL	-	95.12 ± 0.38	87.25 ± 0.25	89.50 ± 0.35
	DANN	-	96.09 ± 0.25	89.50 ± 0.17	73.90 ± 0.43
	MRMI	-	99.61 ± 0.20	99.60 ± 0.05	99.50 ± 0.20
B	DAFD	75.71 ± 1.05	-	79.25 ± 0.83	70.50 ± 2.30
	GFK	92.50 ± 0.36	-	89.00 ± 0.50	76.10 ± 1.40
	TJM	80.54 ± 0.18	-	85.00 ± 0.00	76.00 ± 0.15
	ARTL	77.86 ± 0.95	-	83.75 ± 0.63	73.00 ± 1.20
	DANN	96.07 ± 0.46	-	93.25 ± 0.14	65.70 ± 0.29
	MRMI	99.64 ± 0.25	-	100.00 ± 0.00	99.80 ± 0.05
C	DAFD	74.29 ± 0.78	82.93 ± 1.35	-	72.60 ± 0.45
	GFK	89.29 ± 0.26	95.7 ± 0.31	-	93.20 ± 0.40
	TJM	80.76 ± 0.54	84.88 ± 0.00	-	79.00 ± 0.25
	ARTL	88.93 ± 0.44	80.98 ± 0.36	-	93.60 ± 0.60
	DANN	97.14 ± 0.18	96.59 ± 0.31	-	90.90 ± 0.78
	MRMI	98.86 ± 0.35	100.00 ± 0.00	-	99.95 ± 0.05
D	DAFD	76.43 ± 0.28	76.20 ± 1.35	71.25 ± 0.75	-
	GFK	97.00 ± 0.50	97.5 ± 0.26	98.10 ± 0.65	-
	TJM	95.00 ± 0.36	78.54 ± 0.56	80.75 ± 0.50	-
	ARTL	93.93 ± 0.48	89.76 ± 0.18	92.00 ± 0.58	-
	DANN	91.79 ± 0.84	95.61 ± 0.36	91.00 ± 0.19	-
	MRMI	96.86 ± 0.05	99.75 ± 0.25	100.00 ± 0.00	-

**Table 4:** An ablation study for MRMI: Performances are evaluated on rolling bearing dataset.

Model	MR	MVD	ℓ2-norm	Instance Reweighting	Softmax Regression	KNN	Average Accuracy (%)
MRMI	**√**	**√**	**√**	**√**	**√**		99.46
Without MR		**√**	**√**	**√**	**√**		97.28
Without MR, MVD			**√**	**√**	**√**		93.85
Without MR, MVD, ℓ2-norm				**√**	**√**		89.41
MRMI with KNN	**√**	**√**	**√**	**√**		**√**	98.52

**Table 5:** Gear dataset with sample class imbalanced distribution.

Fault Type	Type 1	Type 2	Type 3	Type 4	Type 5	Total
Category Labels	1	2	3	4	5	
Dataset A	100	40	30	20	10	200
Dataset B	50	15	10	15	10	100
Dataset C	100	100	100	100	100	200

## Data Availability

Not applicable.
